# Prevention of infective endocarditis in developing countries

**DOI:** 10.5830/CVJA-2012-004

**Published:** 2012-07

**Authors:** Breminand Maharaj, Andrew Parrish

**Affiliations:** Department of Therapeutics and Medicines Management, Nelson R Mandela School of Medicine, University of KwaZulu-Natal, Durban, South Africa; Department of Internal Medicine, Walter Sisulu University, Umtata, South Africa

Infective endocarditis (IE) causes substantial morbidity and mortality despite modern antimicrobial chemotherapy and advances in the ability to diagnose and treat complications.^1^,^2^ Prevention of IE is, therefore, very important. Infective endocarditis usually develops following a bacteraemia in individuals with underlying structural cardiac defects. Bacteraemia may occur spontaneously, follow everyday procedures or complicate certain interventions, such as dental extraction.^3^,^4^

In developing countries, IE occurs most frequently in patients with rheumatic heart disease (RHD).^1^,^2^ The first step in the prevention of IE would be to reduce the pool of patients who are susceptible to this infection. This requires the effective implementation of programmes to prevent rheumatic fever and, therefore, RHD.^5^,^6^ Regrettably, this has not happened in developing countries.^5^ Furthermore, prophylaxis against IE has been neglected or seen as a separate issue.

The prevention of both IE and rheumatic fever recurrences should be viewed as part and parcel of the care of a patient with RHD in order to reduce unnecessary morbidity and mortality in patients with RHD in developing countries. RHD should be prioritised in developing countries and a greater emphasis needs to be placed on the simple and cost-effective measures that are currently available to combat RHD in the developing world.^5^-^8^

Many patients with RHD are unaware of the presence of their underlying heart disease and are, therefore, unable to request prophylaxis against IE.^1^,^9^,^10^ School-based screening programmes are beneficial in detecting undiagnosed RHD.^1^,^7^,^9^-^12^ It has been proposed that in developing countries, registered nurses be trained to detect children with cardiac abnormalities, refer them to doctors organising the screening programme for assessment, and thereafter maintain follow up of these patients.^9^ The nurses would be responsible for ensuring secondary prophylaxis against rheumatic fever and prophylaxis against IE. The doctor in such a nurse-orientated primary healthcare service would be responsible for the organisation, monitoring and continuity of such programmes.

Dodu and Bothig stated that in many countries, nurses are trained to recognise certain criteria such as heart murmurs for referral and have proved to be both reliable and efficient in identifying children who need medical examination.^13^ School-based surveys could be made more effective if performed as part of a general-purpose health survey of school children. The ultimate aim should be to incorporate screening surveys into a routine school examination system conducted on a regular basis or to establish such services where they do not exist.

Such school-based surveys can only reach children who attend school. Children who do not attend school belong more often to the poorer, crowded communities where the problem of rheumatic fever/rheumatic heart disease is of greater magnitude. Ultimately, a strategy for the early detection of RHD in childhood that includes non-school goers will have to be developed.

Children identified with RHD or a history of rheumatic fever should be referred for secondary prophylaxis against rheumatic fever as well as prophylaxis against IE. In addition, adults with a history of rheumatic fever, RHD or cardiac valve surgery can be referred. One team could therefore be responsible for the prevention of both rheumatic fever/rheumatic heart disease and IE. Health education programmes should also be designed to motivate such patients and their families to accept secondary prophylaxis against rheumatic fever and prophylaxis against IE on a regular long-term basis and to enlist their co-operation for maintaining a high level of patient compliance.

Antibiotic prophylaxis against IE has been accepted in most countries for many years,^14^-^17^ even though no prospective studies have proven their effectiveness.^16^-^20^ Antimicrobial prophylaxis has been recommended prior to dental extraction in order to prevent post-extraction bacteraemia and the subsequent development of IE. In the study of amoxicillin, clindamycin or chlorhexidine administered prior to dental extraction, none of the treatments eliminated post-extraction bactraemia.^17^ The article by Durack, which evaluated some of the drugs used for prophylaxis prior to dental extraction, confirmed that antimicrobials do not prevent post-extraction bacteraemia.^18^

Recent guidelines have introduced major changes to recommendations for the use of prophylactic antibiotics. The working party of the British Society for Antimicrobial Chemotherapy (BSAC) stated that despite the lack of evidence for prophylactic antibiotics to prevent IE associated with dental procedures, they considered that many clinicians would be reluctant to accept the radical but logical step of withholding antibiotic prophylaxis for dental procedures.^21^ They therefore compromised and recommended prophylaxis for only those patients in whom the risk of developing IE is high and, if infected, would carry a particularly high mortality rate.

The new American Heart Association (AHA) guidelines^22^,^23^ are similar to the BSAC guidelines for dental procedures, but differ in that they do not recommend prophylaxis before gastrointestinal or genitourinary procedures. Neither of these guidelines included RHD as one of the cardiac conditions for which prophylaxis is recommended. The National Institute for Clinical Excellence (NICE) does not recommend antibiotic prophylaxis for patients undergoing dental, gastrointestinal or genitourinary procedures.^20^

The National Essential Drug List Committee prepares and revises standard treatment guidelines for the public sector in South Africa. The Adult Expert Review Group for the hospital level reviewed the literature, including the above guidelines, and acknowledged that clinicians would be concerned with both the exclusion of RHD from the list of cardiac conditions that required prophylaxis and with not giving an antibiotic prior to dental extraction. The Review Group recommended the use of antibiotics prior to certain dental procedures and included acquired valvular heart disease with stenosis or regurgitation as a cardiac condition that requires prophylaxis. Further debate culminating in a concensus position on antibiotic prophyaxis in developing countries is needed.

It should be noted, however, that antibiotic prophylaxis no longer occupies centre stage in the prevention of IE. The BSAC has endorsed the NICE guidelines.^24^ The emphasis for IE causation has shifted from procedure-related bacteraemia to cumulative bacteraemia, i.e. infective endocarditis is more likely to result from frequent exposure to random bacteraemias associated with daily activities such as tooth brushing than with a dental procedure.^3^,^4^,^18^,^20^-^23^ Poor oral hygiene may increase the risk of bacteraemia associated with these everyday procedures.^22^,^25^

A number of authors have stressed good oral health as a far more important preventative measure than chemoprophylaxis against IE.^3^,^26^-^30^ The BSAC stated that good oral hygiene is probably the most important factor in reducing the risk of IE in susceptible individuals, and access to high-quality dental care should be facilitated.^21^

The AHA has stated that the maintenance of optimal oral health and hygiene may reduce the incidence of bacteraemia from daily activities, and it is more important than prophylactic antibiotics for dental procedures to reduce the risk of IE.^23^ NICE has also placed emphasis on the importance of this aspect of prophylaxis.^20^ Improving oral hygiene and reducing or eliminating gingivitis would reduce the incidence of bacteraemia following tooth brushing and the need to extract teeth due to periodontal disease and caries.^25^

The accompanying study on the oral health status of patients with severe RHD who were awaiting cardiac surgery revealed that inadequate attention is being paid to the maintenance of good oral health in these patients (page 336). It is very likely that within the healthcare systems of developing countries, the oral health of patients with less-severe RHD who are not attending specialist cardiac facilities is also suboptimal. Also knowledge regarding the need and measures for prevention of IE among patients is insufficient.^31^-^35^

Care of the oral health of patients at risk of developing IE needs to be improved. Greater awareness of this problem among cardiologists, cardiothoracic surgeons, physicians, paediatricians, medical practitioners and dental surgeons is needed. The results of the study by Bobhate and Pinto indicate that the majority of oral lesions could have been prevented if patients had been informed of the vital importance of preventative dentistry.^36^ Therefore, advice on regular oral care and the maintenance of oral health must be given to all patients at risk of developing IE and their parents (in the case of children).^36^

Furthermore, patients and parents of children need to be given appropriate advice regarding antibiotic prophylaxis, including informing their dentist that they have heart disease that may require antibiotics, before dental procedures, particularly dental extraction, can be done. Patients and parents of children with RHD also need advice on secondary prophylaxis of rheumatic fever.

At the time of diagnosis of a heart lesion that could predispose to IE, a full oral examination, including dental radiography, should be performed. Further examinations at frequent and regular intervals will ensure early diagnosis and treatment of oral lesions, and maintenance of good oral hygiene. It is advisable to issue patients with a warning card on which their cardiac condition, drug therapy, suggested prophylactic measures to be taken before dental manipulations, and name, address and telephone number of the attending doctor is recorded. A medical history should be obtained from every patient before institution of any dental treatment.

Co-operation between doctors and dental surgeons and their support staff, e.g. oral hygienists, would ensure that the oral health needs of every susceptible patient, including patient education, can be catered for more adequately. Supplies such as toothbrushes and toothpaste should be made available to these patients. Social support, including social grants, could assist patients in financial difficulty. The key to protection of susceptible patients is improved oral health education, effective preventative care, oral hygiene instruction and sensible treatment planning. An oral hygienist should form part of the team that cares for patients with RHD.

A number of surveys have revealed that doctors and dentists are familiar with the concept of prophylaxis against IE, are aware of the published recommendations and believe them to be authoritative, yet prophylaxis is not used for many patients for whom it is indicated, and specific recommendations and regimens are not followed.^37^-^40^ The medical and dental curricula need to place appropriate emphasis on all aspects of prophylaxis, including patient education, maintenance of good oral health and antibiotic prophylaxis.

The prevention of IE has been neglected in the past and does not solely concern antibiotic prophylaxis.^21^ There should be a shift in emphasis away from antibiotic prophylaxis prior to dental procedure, towards a greater emphasis on improved access to dental care and oral heath in patients with predisposing cardiac conditions.^22^ The first step in the prevention of IE in developing countries would be to reduce the pool of patients who are susceptible to this infection through implementation of programmes to prevent rheumatic fever.

The second step would be the early identification of at-risk patients, and prompt referral to oral health specialists, e.g. oral hygienists, for comprehensive evaluation and treatment. The third step would be to educate these patients and the parents of children on the need for maintaining optimal oral health and about antibiotic prophylaxis for IE. The fourth step would be to integrate IE prophylaxis into rheumatic fever/rheumatic heart disease prevention programmes and provide more holistic care of patients with rheumatic heart disease.

Accordingly, a comprehensive prevention programme, which incorporates the secondary prevention of rheumatic fever and the prevention of IE, is urgently needed in developing countries. Optimal oral healthcare, in conjunction with the judicious use of antibiotic prophylaxis for prevention of IE, should serve to reduce the significant morbidity and mortality associated with this infection. It is hoped that the care of patients at risk of developing infective endocarditis will thus be improved.
